# Characterization of the complete chloroplast genome of *Thalictrum atriplex* (Ranunculaceae)

**DOI:** 10.1080/23802359.2026.2722744

**Published:** 2026-08-28

**Authors:** Shihao Jiang, Qinyi Wang, Xingyu Du, Yanru Zhang, Yi Tang, Ying Huang, Xia Pan, Yan Chen, Liang Pang, Zhixi Fu, Xianhua Gu

**Affiliations:** ^a^ Ministry of Education, Key Laboratory of Land Resources Evaluation and Monitoring in Southwest, Sichuan Normal University, Chengdu, China; ^b^ College of Life Sciences, Sichuan Normal University, Chengdu, China; ^c^ Leshan Eco-environmental Monitoring Center Station of Sichuan Province, Leshan, China; ^d^ Xizang Shengyuan Environmental Engineering Co., Ltd., Lhasa, China; ^e^ Sichuan Guoce Testing Technology Co., Ltd., Chengdu, China

**Keywords:** *Thalictrum atriplex*, phylogenetic relationship, coding sequences, chloroplast genome

## Abstract

This study determined the chloroplast genome of *Thalictrum atriplex*, which is 155,984 base pairs (bp) in length, including one large single-copy (LSC) region of 85,422 bp, one small single-copy (SSC) region of 17,594 bp, and two inverted repeat (IR) regions of equal length (26,484 bp each). The total guanine–cytosine (GC) content was 38.35%. A total of 127 genes were identified, including 83 protein-coding genes, eight ribosomal RNA (rRNA) genes, and 37 transfer RNA (tRNA) genes. Phylogenetic analysis revealed that *T. atriplex* and *T. finetii* are closely related, forming a monophyletic clade, which provides a basis for the phylogenetic study and resource utilization of *T. atriplex*.

## Introduction

The genus *Thalictrum* is an important genus in the family Ranunculaceae, with approximately 200 species, making it the largest genus in the family. It is widely distributed in the Northern Hemisphere (Ling et al. 2024; Xiang et al. 2026). In China, its distribution is mainly in the southwest, northwest, and mountainous areas of North China (Chen et al. 2024). Ethnobotanical studies have shown that *Thalictrum* possesses a wide range of pharmacological activities and is widely used to treat rheumatism, trauma, joint pain, indigestion, and other ailments (Chen et al. 2003; Singh et al. 2023). The species of *Thalictrum atriplex* Finet & Gagnep. (1903) is mainly distributed in Jiangxi, Chongqing, Sichuan, Yunnan, Tibet, Shaanxi, Gansu, and Qinghai provinces in China, growing on grassy slopes, forest edges, or in sparse forests at altitudes of 2300–3600 m (Meng et al. 2021).

Compared to nuclear and mitochondrial genomes, chloroplast genomes are conserved and compact with moderate nucleotide substitution rates, making them ideal molecular vectors for plant phylogenetic analysis (Jansen et al. 2007; Daniell et al. 2016). Most angiosperm chloroplast genomes exhibit a typical four-part circular structure, including a pair of inverted repeat (IR) regions, a large single-copy (LSC) region, and a small single-copy (SSC) region, with the IR region separating the two single-copy regions (Wu et al. 2021; Yan et al. 2023).

This study sequenced, assembled, annotated, and characterized the complete chloroplast genome of *T. atriplex*. It also explored the phylogenetic relationships of *T. atriplex* within the genus by combining data from closely related species, providing basic data support for the phylogeny of *Thalictrum* plants.

## Materials and methods

### Sampling, extraction, and genome sequencing

**Figure 1.**
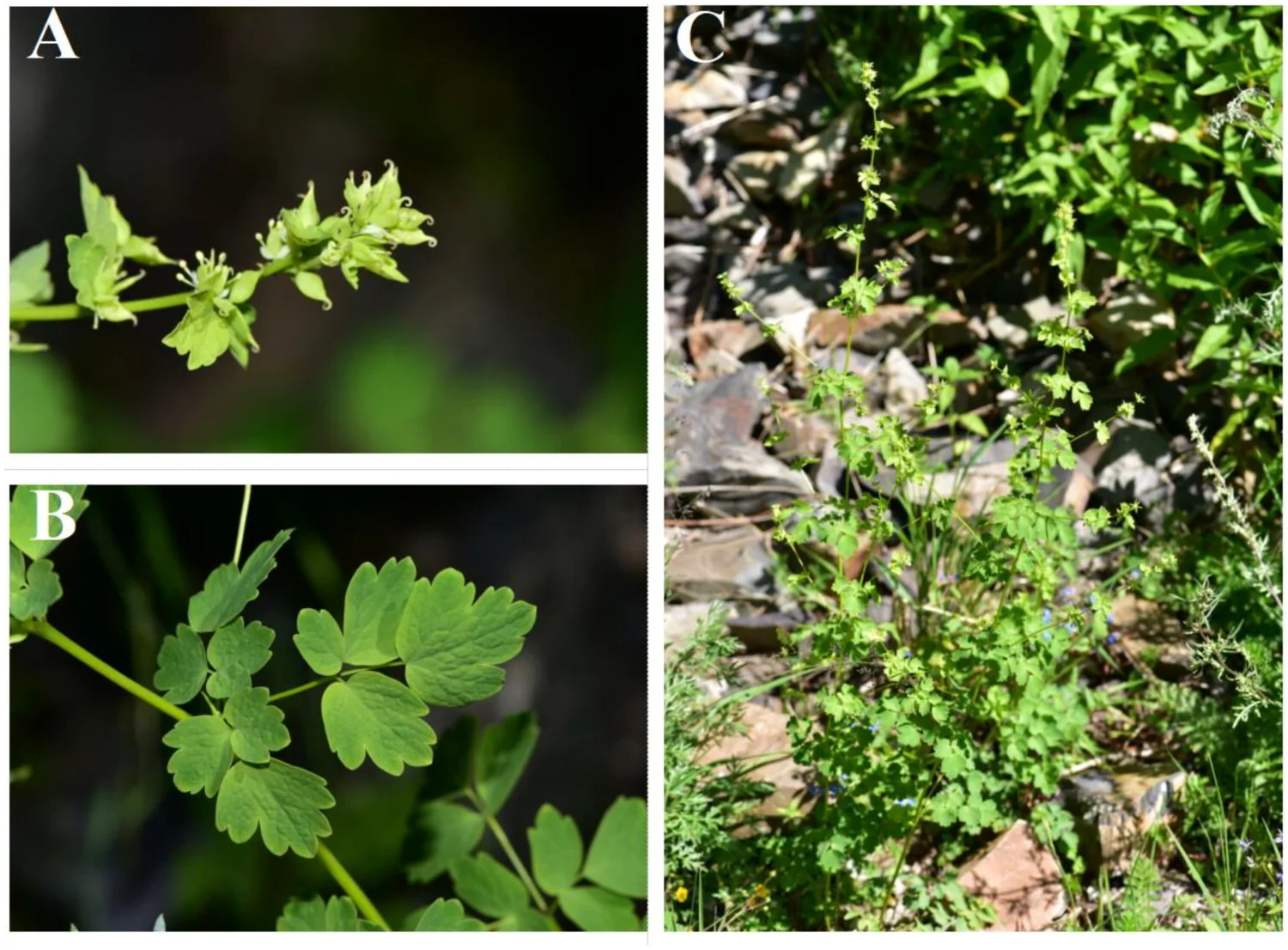
Photos of *T. atriplex* taken by Meng Dechang, with no copyright issues. (A) The spike-like inflorescence in the distance; (B) the leaves as seen up close; (C) the entire plant. This plant is a perennial herb, glabrous. The stem is generally 80 cm tall. The lower leaves have long petioles; the leaflets are 0.8–2.2 cm long. The panicles are narrow and long, resembling racemes; the flowers are relatively dense; the pedicels are 1–5 mm long; the sepals are 4, white, caducous, elliptic, 2.5–3.5 mm long; the stamens are 7–10, the filaments are club-shaped at the upper part and linear at the lower part, the anthers are elliptic; the carpels are 4–5, the styles are curved backwards.

This study collected *T. atriplex* samples from a wild population in Seda County, Ganzi Tibetan Autonomous Prefecture, Sichuan Province, China (32.040°N, 100.511°E) (Figure 1). Voucher specimens were identified by Dr. Zhixi Fu and are deposited at the Herbarium of Sichuan Normal University (SCNU, https://bio.sicnu.edu.cn/; Contact person: Dr. Zhixi Fu, email: fuzx2017@sicnu.edu.cn). The voucher specimen number of the species is SDX0477. Fresh leaves were collected and rapidly dried in sealed bags containing silica gel. Total genomic DNA was extracted from leaf tissue using a modified cetyltrimethylammonium bromide (CTAB) method (Allen et al. 2006). Paired-end (PE) sequencing libraries were constructed using the Illumina Paired-End DNA Library Construction Kit (Illumina, San Diego, CA). Library sequencing was performed on the Illumina NovaSeq 6000 platform (Novogene Technology Co., Ltd., Beijing, China), generating 150 base pairs (bp) reads. Raw sequencing data were obtained using the Illumina Genome Analyzer (Illumina, San Diego, CA).

### Assembly and annotation of chloroplast genome

**Figure 2.**
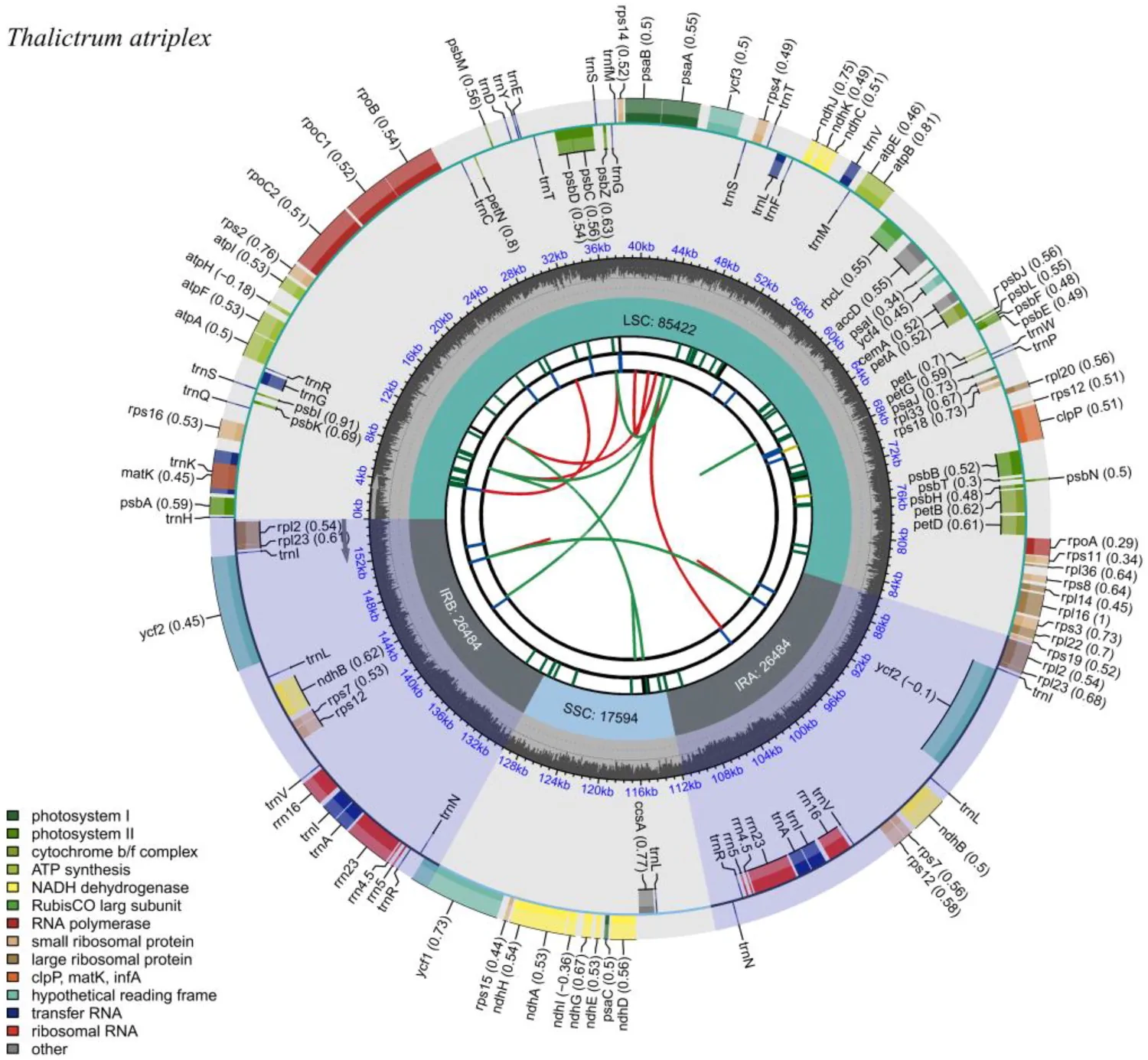
A schematic diagram of the overall chloroplast genome characteristics of *T. atriplex.*

Chloroplast genomes were assembled using SPAdes v3.15.1-Linux software with default parameters (Bankevich et al. 2012). This study used BWA v0.7.17 to generate depth of coverage (minimum depth 505×; maximum depth 1815×; average depth 1327.74×) (https://www.protocols.io/view/generating-sequencing-depth-and-coverage-map-for-o-4r3l27jkxg1y/v1) (Figure S1). The quality and circularity of the assembled genomes were assessed and visualized using Bandage software (Wick et al. 2015). The annotation result was visualized using the CPGview program (Liu et al. 2023) (Figures S2 and S3). Genome annotation was performed using the Plastid Genome Annotator (PGA) workflow (Qu et al. 2019), and the annotation results were manually checked and corrected using Geneious R11 (Kearse et al. 2012). A complete annotated chloroplast genome map was constructed using the CPGview online tool (Liu et al. 2023) (Figure 2).

### Phylogenetic analysis

To conduct phylogenetic analysis, we retrieved chloroplast genome sequences from 19 published *Thalictrum* species from the GenBank database, as well as from two closely related genera (*Paraquilegia* and *Leptopyrum*) that are structurally preserved and have complete data (Zhao et al. 2020; Zoclanclounon et al. 2023). Phylogenetic analysis was performed on a dataset of complete chloroplast genomes from 21 plant species using maximum-likelihood (ML) implemented in IQ-TREE. All sequences were aligned using MAFFT v7.526 (Katoh and Standley 2013). Maximum-likelihood tree construction was performed on the GTRGAMMA model using IQ-TREE (Stamatakis 2014), and 1000 bootstrap replications were performed (Miller et al. 2010).

## Results

### Genome structure analysis

The full-length chloroplast genome of *T. atriplex* is 155,984 bp, exhibiting a typical tetrad structure (Figure 2). A pair of 26,484 bp IR regions (inverted repeat a, IRa; inverted repeat b, IRb) separate the 85,422 bp LSC region and the 17,594 bp SSC region, indicating a guanine–cytosine (GC) content of 38.35%. A total of 127 genes (110 unique genes) were annotated, including 83 protein-coding genes, eight ribosomal RNA (rRNA) genes, and 37 transfer RNA (tRNA) genes (Table S1). Its 5′ exon is located in a LSC region, whereas the 3′ exon is situated within an IR region (Figure S2). The genome contains 17 cis-splicing genes (Figure S3). The physical distance between the two exons is approximately 70 kb. Trans-splicing is required to form mature messenger RNA (mRNA). Its complete genome sequence is deposited in GenBank with accession number PX686334.

### Phylogenetic analysis

**Figure 3.**
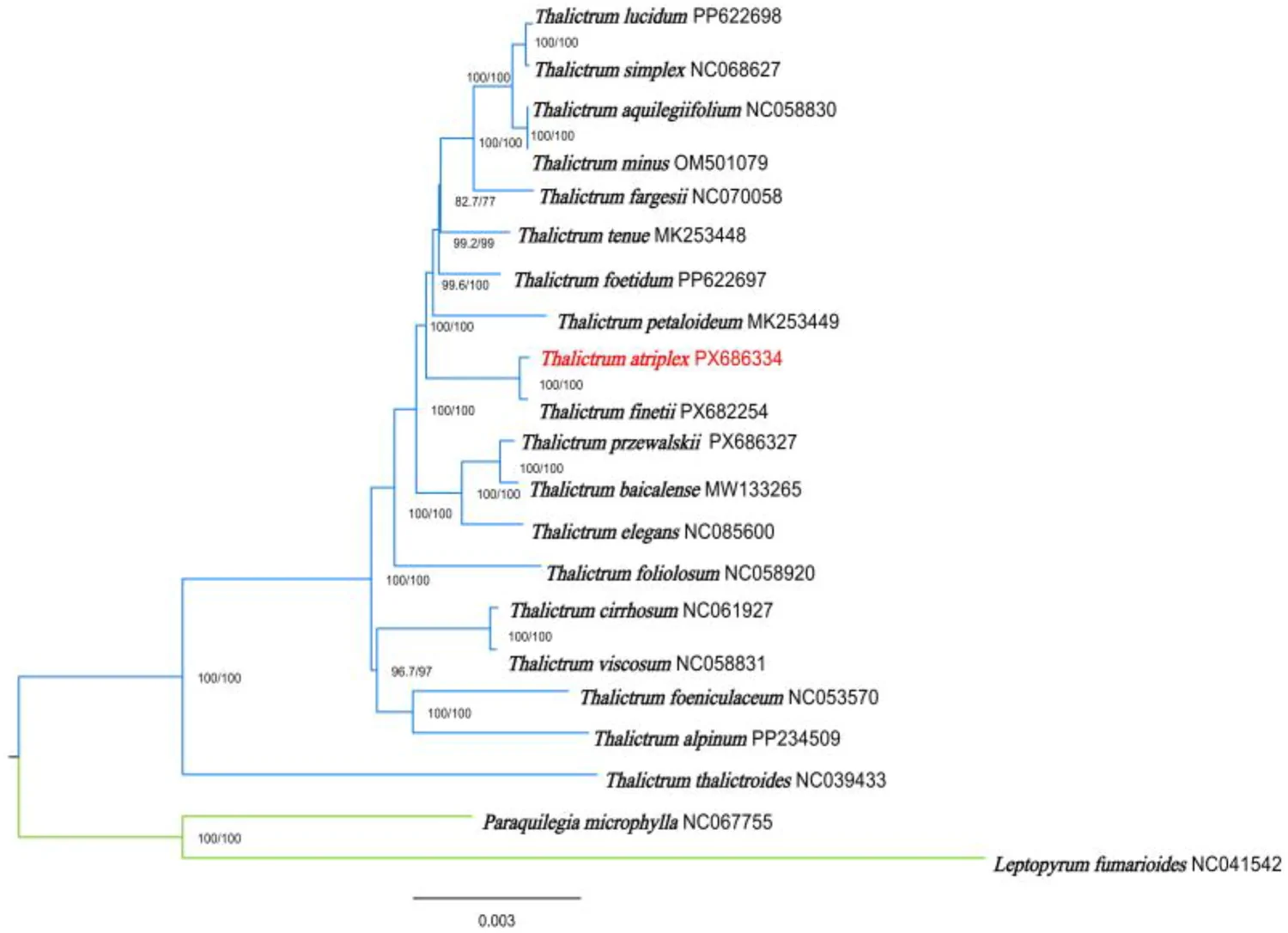
The best maximum-likelihood (ML) phylogenetic tree inferred from 21 chloroplast genomes (bootstrap values are labeled on the branches). Species marked in red were newly sequenced in this study. The sequences used include: *T. simplex* NC068627.1 (Xiang et al. 2022), *T. aquilegiifolium* NC058830.1 (Xiang et al. 2022), *T. minus* var. *hypoleucum* OM501079.1 (Xiang et al. 2022), *T. fargesii* NC070058.1 (Pu et al. 2022), *T. atriplex* PX686334.1 (Meng et al. 2021; Zeng et al. 2022), *T. finetii* PX682254.1 (unpublished), *T. viscosum* NC058831.1, *T. thalictroides* NC039433.1 (unpublished), *T. baicalense* MW133265.1 (He et al. 2021), *T. przewalskii* PX686327.1 (the sequenced species), *T. lucidum* PP622698.1, *T. foetidum* PP622697.1 (Ding et al. 2019), *T. alpinum* PP234509.1 (unpublished), *T. elegans* NC085600.1 (Cheng et al. 2024), *T. cirrhosum* NC061927.1, *T. foliolosum* NC058920.1 (Pu et al. 2022), *T. foeniculaceum* NC_053570.1 (Lin et al. 2021), *T. petaloideum* MK253449.1 (Chae et al. 2025), *T. tenue* MK253448.1 (Xu et al. 2020), *Paraquilegia microphylla* NC067755.1 (unpublished), and *Leptopyrum fumarioides* NC041542.1 (unpublished).

The results showed that *T. atriplex* and *T. finetii* constitute a monophyletic clade (100/100 support), and their branch forms a monophyletic clade with *T. petaloideum* L. 1762 (100/100 support). This phylogenetic tree will contribute to further research on *T. atriplex* (Figure 3).

## Discussion and conclusions

The chloroplast genome structure of *T. atriplex* is relatively stable, consisting of LSC, SSC, and IR regions (IRa/IRb) (Zhu et al. 2016; Wu et al. 2021; Gu et al. 2024) (Figure 2). In terms of length, the chloroplast genomes of *T. atriplex*, *T. fargesii*, *T. finetii*, and *T. petaloideum* are similar, ranging from 155,876 bp to 156,009 bp. The LSC region ranges from 85,326 bp to 85,464 bp, the IR region from 26,479 bp to 26,484 bp, and the SSC region from 17,576 bp to 17,594 bp (Table S2). The GC content of these four *Thalictrum* species is also very similar, around 38.5%. The number and content of genes are very close, consistent with previous studies (Wicke et al. 2011).

The results showed that all *Thalictrum* species formed a highly supported monophyletic clade, with *T. atriplex* and *T. finetii* forming a clear monophyletic clade relationship. This result is consistent with previous studies based on chloroplast fragments (Wicke et al. 2011) (Table S3). This study provides data support for our future research and conservation of *Thalictrum* species.

## Supplementary Material

Supplemental Material

## Data Availability

The genome sequence data that support the findings of this study are openly available in National Center for Biotechnology Information (NCBI) GenBank database at (https://www.ncbi.nlm.nih.gov/) under accession no. PX686334 (https://www.ncbi.nlm.nih.gov/nuccore/PX686334). The associated BioProject, SRA, and Bio Sample numbers are PRJNA1455079, SRR38184253 (Illumina), and SAMN57342757, respectively.
