# Supplementary material for: Characterization of the complete chloroplast genome of Thalictrum atriplex (Ranunculaceae)
Source: Mitochondrial DNA B Resour. 2026 Aug 28;11(9):1080–4. doi: 10.1080/23802359.2026.2722744 (PMC13528116; doi:10.1080/23802359.2026.2722744)
Supplement: Supplementary file 1 [file TMDN_A_2722744_SM6138.zip › Figure S2.doc]

**Figure S2.** Schematic diagram of trans-spliced genes in the chloroplast genome of *Thalictrum atriplex*.
